# Locked in and left out: the "prison penalty" for implementation of evidence-based interventions

**DOI:** 10.1186/s43058-024-00573-0

**Published:** 2024-04-09

**Authors:** Justin Berk, Hannah E. Frank, Mari-Lynn Drainoni

**Affiliations:** 1 Departments of Medicine and Pediatrics, Alpert Medical School at Brown University, 245 Chapman St;, RI Providence, 02906 USA; 2 Department of Psychiatry and Human Behavior, Brown Research On Implementation and Dissemination to Guide Evidence Use (BRIDGE) Program, Division of Biology and Medicine, The Warren Alpert Medical School of Brown University, Providence, USA; 3 Department of Medicine, Section of Infectious Diseases, Boston University Chobanian & Avedisian School of Medicine, 801 Massachusetts Ave, Room 2014, MA Boston, 02118 USA; 4 Department of Health Law Policy & Management, Boston University School of Public Health, 801 Massachusetts Ave, Room 2014, MA Boston, 02118 USA; 5 Department of Medicine, Evans Center for Implementation and Improvement Sciences, Boston University Chobanian & Avedisian School of Medicine, 801 Massachusetts Ave, Room 2014, MA Boston, 02118 USA

## Abstract

**Background:**

While the broader medical community grapples with the widely accepted notion that it takes an average of 17 years for research evidence to be incorporated into clinical practice, the implementation of evidence-based interventions in carceral settings (i.e., jails and prisons) faces longer delays, exacerbating health disparities.

**Main body:**

The “prison implementation penalty” describes the significant delay in and limited adoption of evidence-based healthcare practices in carceral settings. We explore the complex challenges of implementing evidence-based interventions in jails and prisons, environments where healthcare often plays a secondary role under security and discipline. We use specific frameworks to highlight the unique barriers within these settings and propose potential implementation strategies. These challenges have broad implications for health equity due to the disproportionate impact on the marginalized groups affected by mass incarceration. Implementation science has potential to mitigate these disparities.

**Conclusion:**

Bridging the gap between healthcare evidence and practice in carceral settings offers a public health opportunity. Implementation science offers a unique role in improving healthcare standards and reducing health inequities in this environment.

Contributions to the literature

- The “prison implementation penalty” term highlights the delay in adopting evidence-based interventions in carceral settings, underscoring the unique cultural and logistical barriers in jails and prisons that extend the commonly-cited 17-year lag between evidence and practice.
- Emerging research within implementation science seeks to address these challenges by identifying contextual barriers, tailoring implementation strategies, and evaluating implementation outcomes using well-established frameworks.
- Implementation science holds substantial potential to advance health equity by engaging a marginalized community impacted by the criminal legal system.

## Background

Among the complexity of healthcare services delivery, carceral settings—jails and prisons—create some of the most challenging environments for the implementation of evidence-based practices. The broader medical community grapples with the notion that it takes an average of 17 years for research evidence to be incorporated into clinical practice [1]. Even then, fewer than 1 in 5 evidence-based practices ultimately make it into routine clinical practice [1]. While the field of implementation science has sought to reduce this research-practice gap, the current state of the United States (US) carceral system only exacerbates this delay in implementation. The extended lag in adopting evidence-based healthcare practices and the limited uptake of such interventions in US prisons and jails is what we term the “prison implementation penalty.”

As incarcerated individuals are constitutionally entitled to healthcare [2], this delay represents a failure to uphold legal mandates and exacerbates health disparities [3] among one of the most marginalized populations. In the field of bioethics and human rights, the concept of “Equivalence of Care” argues for parity between carceral healthcare systems and community healthcare systems and is a core part of the United Nations Mandela Rules for a minimum standard for the treatment in prison. Such a standard, however, frequently goes unmet in the US carceral system [4]. The field of implementation science offers a viable approach to addressing these deficiencies and striving towards the equivalence of care ideal.

Through this Commentary, we describe the prison implementation penalty and underscore the pressing need for the principles of implementation science to bridge the chasm between evidence and practice in these unique settings.

## The prison implementation penalty

Carceral settings face distinct barriers when it comes to implementing evidence-based interventions (EBI). The healthcare standards in jails and prisons often lag behind community settings, and many interventions may never make it to carceral settings. While there are instances in which care for incarcerated individuals meets community standards, more often there are stark examples where care has been described as “a barren wasteland of medical care” [5]. Even the provision of basic healthcare services in the carceral setting can pose significant hurdles.

Healthcare improvements have been described as “piecemeal” and typically with a focus only on reaching minimum established standards, rather than implementing new EBIs [6]. Robust data are lacking but suggest substantial delays or lack of access entirely to mental healthcare, palliative care for geriatric patients [6], and cancer screenings and treatment [7]. This gap between carceral care and routine care demonstrates the additional “penalty” that incarceration imposes. Indeed, litigation remains a mainstay of enforcing minimal standards of community care in jails and prisons [6]. Maintaining the latest medical practices can be a challenging task in these settings; introduction of new treatments, interventions, and guidelines often encounter significant delay.

Carceral facilities stand out among other “inner settings” in implementation science and work in these settings has “not kept pace with advancements in implementation science research and methodology” [8]. Unlike traditional healthcare institutions whose primary mandate is to provide healthcare, jails and prisons primarily serve as establishments for law enforcement, with healthcare delivery often being a secondary objective. While other settings like schools, homeless shelters, street medicine, and assisted living facilities also provide healthcare outside of conventional environments, their primary missions may align with public health goals. Meanwhile, the use of incarceration as a societal response can have direct and detrimental public health implications. Indeed, incarceration often results from racist policies that disproportionately affect communities of color [9]. The values purportedly upheld in carceral settings—security, discipline, and routine—can mask deeper systemic issues of oppression. The conflict between these values and the principles of quality healthcare—such as quality improvement, efficiency, patient satisfaction, and shared decision-making—contributes significantly to the delay in implementing and disseminating EBIs, leading to the “prison implementation penalty.”

## A case example: medications for opioid use disorder in jails and prisons

To better understand how the prison implementation penalty is actualized, we offer a case example from the field of addiction health services practice in carceral settings. Medication for opioid use disorder (MOUD) has been acknowledged as a gold-standard treatment in the community for decades [10]. A number of institutions have demonstrated success in adopting MOUD programs into the carceral setting and in substantially reducing overdose mortality risk [10]. Yet, despite court rulings, executive orders, and legislative mandates, over two-thirds of jails still do not offer any form of OUD treatment, forcing patients to remain untreated or undergo withdrawal if they were receiving community treatment [11]. Moreover, some facilities that do offer treatment provide limited treatment options (e.g., injectable naltrexone only).

Previous research has identified specific implementation barriers in carceral settings that may prevent intervention implementation, may hinder its fidelity, and/or may create a delay in implementation. For example, lack of healthcare funding and limited workforce development in corrections are often cited as challenges to service delivery and expanding programming, including for MOUD programs [12]. Without funding, many MOUD programs in jail or prison settings never ever begin. Unfunded mandates for expansion of MOUD, however, have been put into place, and so, this is not the only contextual determinant.

Other identified barriers hinder the fidelity of newly introduced interventions and cause challenges to successful implementation. Facility culture, staff perceptions, high patient turnover, and the unique physical infrastructure of prisons (designed for surveillance, not healthcare delivery) are identified examples [12, 13]. This may result in limited treatment options (e.g., increased uptake of less evidence-based MOUD formulations like injectable naltrexone).

Some barriers may exacerbate a delay in implementing evidence-based interventions. For instance, physical movement of individuals within a facility, coordination with other government agencies, and the need to identify institutional champions have been identified barriers in prior MOUD research [12]. These extra steps may delay implementation, particularly in organizational settings that, by design, prioritize security over medical. Other facilitators and barriers likely go unnoticed given the limited implementation science research occurring in these settings.

Treatment delay to such a high-risk population can be detrimental. A recent state-wide study suggested almost 50% of opioid overdose mortalities occurred in individuals with recent exposure to the criminal legal system [14]. Expanding evidence-based treatment in the US carceral system has potential to be a core intervention in addressing the national opioid overdose crisis.

## Implementation science frameworks for the health services in jails and prisons

How can implementation science help? Implementation science can determine and adapt key factors that influence healthcare delivery in unique settings. These include identifying the contextual determinants of delivery in carceral settings, tailoring implementation strategies, and assessing implementation outcomes (e.g., fidelity, acceptability) to meet the unique needs of jails and prisons. A recent systematic review acknowledged the relatively limited literature on implementation science applied to the US carceral system. Although some studies have applied robust frameworks like the Consolidated Framework for Implementation Research (CFIR), ERIC, and Proctor’s Taxonomy of Implementation Outcomes, there is not yet a consistent application of these frameworks across the field [8].

### Contextual determinants

**Fig. 1:**
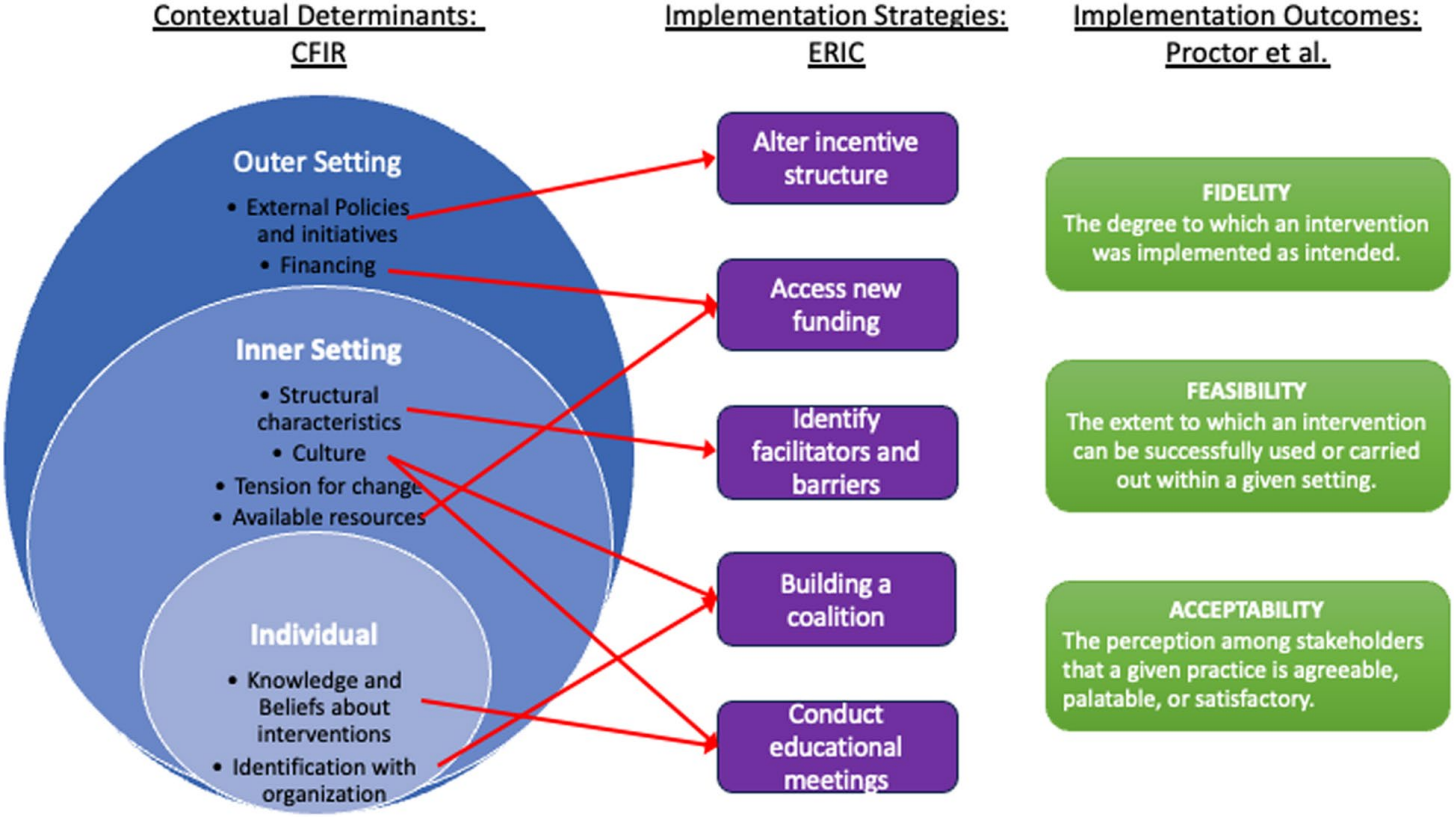
Example determinants, strategies, and outcomes in carceral settings using selected implementation science frameworks

The application of an implementation framework can facilitate a comprehensive understating of the prison implementation penalty. For instance, the CFIR framework can offer insights into the multi-level determinants that impact the implementation of healthcare interventions in these settings. This holistic view emphasizes the need for alignment across various levels, including organizational culture and regulatory environments, to ensure successful implementation. As an example, Fig. 1 illustrates how the CFIR can be used to visualize the barriers to implementation, emphasizing the importance of considering multi-level factors in developing effective strategies for healthcare delivery in jails and prisons.

### Implementation strategies

Implementation science frameworks can leverage the contextual determinants of the prison implementation penalty to guide further research and practice strategies to overcome the barriers. The Expert Recommendations for Implementing Change (ERIC) project utilized an expert panel to come to a consensus on a compilation of 73 implementation strategies that can be used in isolation or combination [15] to address specific implementation barriers. These strategies, designed to be used either in isolation or in combination, can be tailored to address specific implementation barriers in various settings, including carceral environments.

In carceral settings, several ERIC strategies are particularly relevant (see Fig. 1). For instance, “building a coalition” among health services administrators and security personnel can help bridge the gap between healthcare needs and security concerns and partner with external policy-makers to help address organizational and cultural barriers. “Conduct educational meetings” can help address knowledge or beliefs around interventions and affect facility culture. “Access new funding,” while an obvious strategy to address cost and financing concerns, can include seeking opioid settlement funds, public health investments, and utilizing other new policies like the Medicaid 1115 waiver to allow Medicaid coverage for patients while incarcerated.

### Implementation outcomes

Outcome models, such as Proctor’s Taxonomy of Implementation Outcomes, can help research move beyond solely measuring treatment outcomes and include fidelity, acceptability, and adoption in the US carceral system [8]. Commonly used evaluative models (e.g., RE-AIM) could also be applied to evaluate how to best align carceral healthcare delivery to the most evidence-based practices. Process improvement models have also found success in unique environments (e.g., NIATx collaborative learning strategy used to improve delivery logistics of MOUD in Ukraine during the war [16]). By applying these targeted strategies and frameworks, the challenges identified through implementation science can be effectively addressed, enhancing the quality and efficacy of healthcare delivery in jails and prisons.

### Community engagement and equity

Countless implementation science frameworks emphasize the critical role of community engagement and health equity when delivering EBIs (e.g., ERIC strategy: “involve patients/consumers and family members”) [17]. This requires particular attention in carceral settings where community members’ voices have historically been underrepresented. To successfully and equitably implement EBIs in carceral settings, community engagement must be at the forefront and include the voices of incarcerated individuals, in addition to public health experts, government officials, and jail/prison employees [14].

## Potential impact of implementation science in carceral settings

Carceral facilities offer a unique opportunity to efficiently address the pressing health needs of a marginalized patient population. The US trend of mass incarceration over the past 4 decades has dramatically affected people of color. People with disabilities are disproportionately incarcerated, as are transgender and sexual minority persons [18]. Mental health disorders are common: US jails hold 10 times more individuals with mental health diagnoses than state psychiatric hospitals [19]. Incarcerated individuals are also disproportionately affected by chronic diseases, addiction, and communicable diseases. Incarceration accelerates aging, decreases life expectancy, and exacerbates self-harm [20]. Thus, to address public health priorities and to improve health equity, jails and prisons provide a high target environment for intervention. The positive effects of this work resonate beyond the prison facilities, benefiting families, partners, and, importantly, broader communities—particularly as over 95% of incarcerated individuals ultimately return to their neighborhoods and therefore play a pivotal role in public health [20].

Implementation science methods can help to accelerate the translation of research into practice and potentially reduce the harms of such punitive systems. However, despite the promise of implementation science to address the notable inequities in receipt of evidence-based care among incarcerated people, the incorporation of implementation science in carceral settings remains a relatively nascent field with untapped potential [19].

Newer funding mechanisms that aim to integrate implementation science into supported research (e.g., the National Institute of Drug Abuse (NIDA)-funded Justice Community Opioid Innovation Network (JCOIN)) which can address a critical research gap on improving healthcare in jails and prisons.

Based on the unique medical challenges of the incarcerated population, future implementation science work can prioritize several key areas to address the unique healthcare needs within carceral settings:

1. Addressing the high prevalence needs of incarcerated patients including mental health disorders, addiction, sequalae of drug use (e.g., HIV, hepatitis C), and skin/soft tissue infections. This also involves focusing on populations that are disproportionately incarcerated, for instance, by providing gender-affirming care to transgender patients.
2. Enhancing basic primary care services and chronic care management (e.g., hypertension, diabetes) for patient populations that typically have limited access to healthcare services.
3. Improving health maintenance uptake, including vaccinations and cancer screenings.
4. Addressing the social determinants of health that significantly impact incarcerated individuals.
5. Providing specialized care for geriatric populations in carceral settings, who not only face the usual challenges of aging but also experience accelerated aging, greater complications, and have fewer resources within a system that demands a higher standard of activities of daily living.

## The challenges of research and program evaluation in prisons

Addressing the importance of implementation science in carceral settings also requires acknowledging the substantial barriers to conducting research in these environments.

The history of coercion and unethical research practices involving incarcerated individuals necessitates stringent ethical safeguards. These protections, while crucial, add layers of complexity to carceral research programs. For example, there is the logistical hurdle of navigating the prison system’s bureaucracy to ensure good clinical practice and adhere to ethical research standards. There can often be a tension between researchers and prison staff who may feel challenged by public health criticisms of carceral facilities. The individuals who are often responsible for real-world implementation of healthcare services or facility operations may view research activities as disruptive or misaligned with their administrative priorities. This tension can impede the research process. Addressing these challenges requires a nuanced approach that respects the ethical considerations unique to carceral settings while fostering collaboration between researchers and prison staff.

## Conclusion

The “prison implementation penalty” highlights existing gaps in public health and underscores a call for targeted interventions. Policymakers, researchers, healthcare professionals, and administrators can collaboratively harness the tools of implementation science to mitigate the prison implementation penalty and enhance healthcare for a marginalized group. This can include integrating greater measurement implementation-related outcomes in carceral settings. Future clinical trials that include justice-impacted individuals can employ hybrid effectiveness-implementation study designs to ensure a greater understanding of the context of care delivery.

By promoting timely and efficient uptake of EBI, leaders can not only uphold the constitutional right to healthcare for people who are incarcerated but also meaningfully address the broader health inequities that permeate these communities. The strategic use of implementation science within jails and prisons not only can help incarcerated individuals before returning to their community but can also concurrently chip away at entrenched health disparities within society. Even with the integration of implementation science, however, structural oppression and unethical systems that marginalize populations and perpetuate systemic harm require more than incremental improvements. While implementation science can improve care, broader reforms and systemic changes of the “outer setting” are essential for achieving health equity.

## Data Availability

Not applicable.

## Abbreviations

EBI: Evidence-based interventions
CFIR: Consolidated Framework for Implementation Research
EPIS: Exploration, Preparation, Implementation, Sustainment
MOUD: Medication for opioid use disorder
ERIC: Expert Recommendations for Implementing Change
NIDA: National Institute of Drug Abuse
JCOIN: Justice Community Opioid Innovation Network
CJ-DATS: Criminal Justice Drug Abuse Treatment
RE-AIM: Reach, Effectiveness, Adoption, Implementation, Maintenance
NIATx: Network for the Improvement of Addiction Treatment
